# *Clostridioides difficile* infection in a skilled nursing facility (SNF): cost savings of an automated, standardized probiotic antimicrobial stewardship programme (ASP) policy

**DOI:** 10.1093/jacamr/dlad102

**Published:** 2023-09-06

**Authors:** Bridget Olson, Noam Ship, Michael L Butera, Kenneth Warm, Roger Oen, John Howard

**Affiliations:** Department of Pharmacy, Sharp Coronado Hospital & Villa Long Term Care, Sharp HealthCare, 250 Prospect Place, Coronado, CA 92118, USA; Research and Development, Bio-K Plus International Inc., 495 Armand-Frappier Boulevard, Laval, Quebec H7V4B3, Canada; Medical Staff, Sharp Coronado Hospital & Villa Long Term Care, Sharp HealthCare, 250 Prospect Place, Coronado, CA 92118, USA; Medical Staff, Sharp Coronado Hospital & Villa Long Term Care, Sharp HealthCare, 250 Prospect Place, Coronado, CA 92118, USA; Medical Staff, Sharp Coronado Hospital & Villa Long Term Care, Sharp HealthCare, 250 Prospect Place, Coronado, CA 92118, USA; Department of Pharmacy, Sharp Coronado Hospital & Villa Long Term Care, Sharp HealthCare, 250 Prospect Place, Coronado, CA 92118, USA

## Abstract

**Background:**

With multiple comorbidities and frequent exposures to antibiotics, patients in skilled nursing facilities (SNFs) are much more vulnerable to healthcare-acquired infections. We conducted a quality-improvement, retrospective analysis of all patients with *Clostridioides difficile* infection (CDI) from 2009 to 2021 at an SNF. Probiotics were initially added to a bundle of antimicrobial stewardship programme (ASP) CDI prevention strategies. Formulations and durations of probiotics were standardized for both oral and enteral administration. To reach all eligible patients, an ASP probiotic policy provided probiotics with every antibiotic course.

**Objectives:**

To assess the value of providing probiotic therapy to SNF patients at risk for CDI.

**Patients and methods:**

Patients receiving oral or enteral feeding with antibiotics ordered were eligible to receive probiotics. The incremental cost of CDI prevention, treatment and related care were calculated and compared for each phase of probiotic policy change and feeding type. ASP records for the oral probiotic and level of treatment were used in modelling the cost-effectiveness.

**Results:**

From quality improvement initiatives aimed at preventing facility-onset (FO) CDI, to ASP policies, probiotic formulations and delegation of ordering authority, the days of acute care treatment required was significantly reduced over the different phases of implementation [152 to 48, OR = 0.22 (0.16–0.31) to 4, OR = 0.08 (0.03–0.23)] after reducing total CDI from 5.8 to 0.3 cases per 10 000 patient-days. The annual cost of oral probiotics increased from $6019 to $14 652 but the modelled net annual savings for the facility was $72 544–$154 085.

**Conclusions:**

With optimization, the use of probiotics for CDI prevention at an SNF was safe, efficacious and cost-effective.

## Introduction

Approximately one in four *Clostridioides difficile* infections (CDI) in the USA occur in a skilled nursing facility (SNF) or nursing home.^1^ Clinical manifestations can range from mild diarrhoea to toxic megacolon, bowel perforation, septic shock and death. The burden of this nosocomial infection is expectedly high in SNF residents, as many carry the critical risk factors: advanced age, high comorbid illness burden, and frequent antibiotic use.^2,3^

Antibiotics aim to kill pathogenic bacteria, but their use necessarily results in collateral damage to the patient’s own intestinal microbiota.^4,5^*C. difficile* spores are best able to germinate and thrive in this disrupted environment.^6^ Each year, 70% of nursing home residents will be prescribed antibiotics, often unnecessarily,^7^ increasing the risk of CDI. Long-term residency may result in a reservoir of spores and other antibiotic-resistant organisms at the facility, leading to more frequent exposure to nosocomial threats.^2,8^

Despite the prevalence and burden in SNFs, primary prevention of CDI in this setting and its cost-effectiveness are studied less frequently than in the hospital setting.^9–11^ There are ongoing efforts from the US CDC to increase awareness and accountability for responsible antibiotic prescribing and stewardship in nursing homes.^7^ In 2009, our SNF, located in Southern California, initiated a multipronged and adaptive antimicrobial stewardship programme (ASP) to address a notably high burden of CDI, which included the use of probiotics and decreased proton pump inhibitor prescribing with ASP efforts to optimize antimicrobial therapy.^12^ Probiotics are health supplements comprising live microorganisms, which when administered in adequate amounts confer a health benefit on the host.^13^ Taking a specific three-species probiotic alongside antibiotic treatment has been demonstrated to effectively lower the risk of developing CDI in the hospital setting,^14–16^ presumably by temporarily replacing the biochemical functions of the lost intestinal microbes,^17^ and as shown in animal studies, reducing the severity of CDI, and reducing *C. difficile* toxin A and B levels (*Saccharomyces boulardii*), preventing damage to the intestinal mucosa.^18^ Here we provide a 13 year account of the initiation and changes in types of probiotics, and related policies for their administration for primary and recurrent prevention of CDI at an SNF, with an estimate of the cost consequences of this programme.

## Patients and methods

### Setting and intervention

This observational, quality-improvement study took place in a 122 bed SNF, including 12 ventilator-equipped beds, and affiliated with an adjacent, non-profit, 59 bed acute care community hospital in San Diego County, CA, USA. The resident population of this SNF includes those who require short-term rehabilitation post-acute care, such as post-stroke, heart attack, infection or injury, or long-term care (LTC) for multiple comorbidities, needing daily assistance with medical and non-medical needs. The average age of residents in the study was 68 years, with 72% of the residents 65 years of age or older. An ASP co-led by a pharmacist and an infectious disease specialist was started at the acute care hospital in 2009 and implemented a bundle of CDI prevention practices at the SNF, as described previously.^12^ Study subjects included all patients with stools positive for *C. difficile* antigen and toxin, with associated symptoms of infection (*n* = 72). Institutional review board approval was obtained prior to data collection and analysis.

### Phase I (2009–11)

Pharmacists began prospectively reviewing each antibiotic course, with infectious disease physician oversight, for appropriateness of initiation, type of antibiotic and duration of therapy. A lower level of gastric acid suppression was also recommended for patients needing peptic ulcer prophylaxis. Proton pump inhibitors were avoided unless indicated for treatment of active or recent gastrointestinal bleeding.

CDI rates had reached peak levels in 2008 at our facility, with a rate of 8.9 per 10 000 patient-days, and physicians began to prescribe different probiotics, comprised of *Lactobacillus acidophilus* and *Lactobacillus delbrueckii* subsp. *bulgaricus*, 100 million cfu, two to three times a day (Floranex^™^) and/or *S. boulardii* lyo CNCM I-745, 250–500 mg twice daily (Florastor^®^) to patients considered at higher risk of CDI, undergoing longer antibiotic courses (>10 days), or receiving treatment with multiple antibiotics (≥2). The lower doses of both Floranex and Florastor were used more for CDI prophylaxis, and higher doses were favoured by physicians as an addition to CDI treatment, potentially preventing recurrences. Probiotic choice and dosing were not standardized in this first phase. Surgical patients taking antibiotics for prophylaxis for <24 h were not given probiotics.

### Phase II (2012–16)

In 2012, the ASP reviewed probiotic options and available studies for the purpose of standardizing the type and duration of probiotics prescribed. For orally fed patients taking antibiotics, prescribers were encouraged to add a three-species *Lactobacillus* probiotic, two capsules once daily, comprised of *L. acidophilus* CL1285, *Lactobacillus casei* LBC80R and *Lactobacillus rhamnosus* CLR2, 100 billion cfu (Bio-K+^®^ 50 billion) and to administer concurrently with the course of antibiotic therapy, plus 7 days. Enteral tube-fed patients were prescribed a liquid probiotic yogurt twice daily, comprised of *Lacticaseibacillus paracasei* subsp. *paracasei* CNCM I-1518 cfu, 10 billion cfu daily (DanActive^®^) or *L. casei* Shirota, 6.5 billion (Yakult^®^), depending on availability. Gastrostomy tubes were used primarily for enteral probiotic delivery. There was hesitancy giving probiotics through jejunostomy tubes, given the smaller bore of the tubing and possibility of clogging.

In late 2012, the ASP implemented a fever protocol, to improve patient assessments and reporting to physicians, with the goal of averting unnecessary initiation of antibiotics in patients lacking symptoms of bacterial infection.

### Phase III (2017–21)

In late 2016, a protocol was put in place delegating authority to the ASP to add the designated probiotics to all antibiotic courses, to continue during and 7 days beyond the course of antibiotics. The ASP processes and policies established in Phases I and II remained consistent.

Contraindications for probiotic use were established and included severely immunocompromised states and patients unable to receive oral or enteral feedings, or those at increased risk for bacterial translocation and resultant bacteraemia.

### Data collection

Patient profiles maintained by the ASP have allowed for data collection in all patients requiring antibiotic use. The number of facility-wide patient-days, the incidence of CDI diagnosis (primary and recurrent cases) and prescription records for probiotics were compiled from 2009 through to 2021. Data on the annual costs of the probiotics, *L. acidophilus* and *S. boulardii* were only available for 2010 and 2011. Due to the classification of probiotic yogurt as a food product, it was not tracked on the medication administration record, making data unavailable for the utilization and cost of the liquid probiotic yogurt used for CDI prevention in enterally fed patients.

Patient records managed by the ASP for patients with a positive CDI diagnosis indicate the dates of probiotic use, gastric acid suppression, feeding status, and length of stay at the affiliated acute care hospital if transfer was required. Inclusion criteria for our study included all patients on antibiotics with a positive CDI diagnosis. Exclusion criteria included patients with established contraindications to probiotic administration as described in Phase III above. Cost analysis was only performed on orally fed patients, due to the non-availability of enteral probiotic data.

Testing for CDI was performed when patients had unexplained diarrhoea, defined as diarrhoea in the absence of a laxative or bowel stimulant in the previous 48 h. Formed stools were rejected for *C. difficile* testing. The testing criterion was changed in December 2015 from ‘three or more unformed stools within 24 h’. Positive CDIs were detected using a two-step algorithm, beginning in 2009, screening with the C. DIFF QUIK CHEK COMPLETE antigen/toxin assay (TechLab, Blacksburg, VA, USA). When needed, discrepant samples [glutamate dehydrogenase (GDH) positive and toxin A and B negative] were reflexed to PCR testing using the BD GeneOhm Cdiff Assay (Becton, Dickinson and Company, Franklin Lakes, NJ, USA). The molecular assay was changed in 2013 to the Nanosphere Verigene *Clostridium difficile* Nucleic Acid Test (CDF) (Luminex, Austin, TX, USA), then to the ARIES *C. difficile* Nucleic Acid Assay (Luminex) in 2018. In 2017, a comment was added to reports, which included a negative toxin despite a positive antigen/PCR, warning of possible colonization, and to interpret the test result within the context of the clinical findings.

Up to 30 days after discharge from our facility, positive results were considered as acquired from our facility and positive results within 2 days of admission with symptoms of CDI were considered positive on admission and were not attributed to our facility. A primary episode of CDI was defined to be the first identified episode or event in each patient. Recurrent CDI was defined as a repeated case of CDI within 8 weeks of the original CDI event. Leucocytosis (WBC greater than 15 000 cells/μL) or serum creatinine level greater than 1.5 mg/dL were noted as signs of more severe colitis.^8^ CDI was treated as per IDSA guidelines, with oral vancomycin 125 mg, four times daily for 10 days from 2015 on; however, some patients were given vancomycin tapers for prophylaxis during or after periods of long-term antibiotic use, when deemed at high risk for CDI recurrence. Prior to 2015, antibiotic treatment was not standardized, and patients were treated with vancomycin 250 mg orally, four times daily, metronidazole 500 mg orally, three times daily, or a combination of both for 14 days. Vancomycin 500 mg orally, four times daily, plus metronidazole 500 mg IV, every 8 h was given in cases of fulminant CDI, characterized by hypotension or shock, ileus or toxic megacolon. Although two of the newer treatment options for CDI were added to the formulary of our healthcare system, fidaxomicin (2015) and bezlotoxumab (2018), neither was used in any of the nosocomial cases in our SNF. Fidaxomicin was continued in a few cases transferred in from acute care, to complete courses of therapy. Per infection prevention policies, CDI patients were kept isolated, with contact precautions maintained until diarrhoea resolved.^8^ Infection prevention policies were consistent throughout the study period, including terminal cleaning and isolation procedures, up until the COVID-19 pandemic of 2020–21 changed not only personal protective equipment requirements, but also prompted extended disinfecting procedures, with comprehensive and ongoing training of nursing and cleaning staff, in addition to restrictions on patient visitations, mobility and communal exposure during this time.

### Cost model

**Figure 1.**
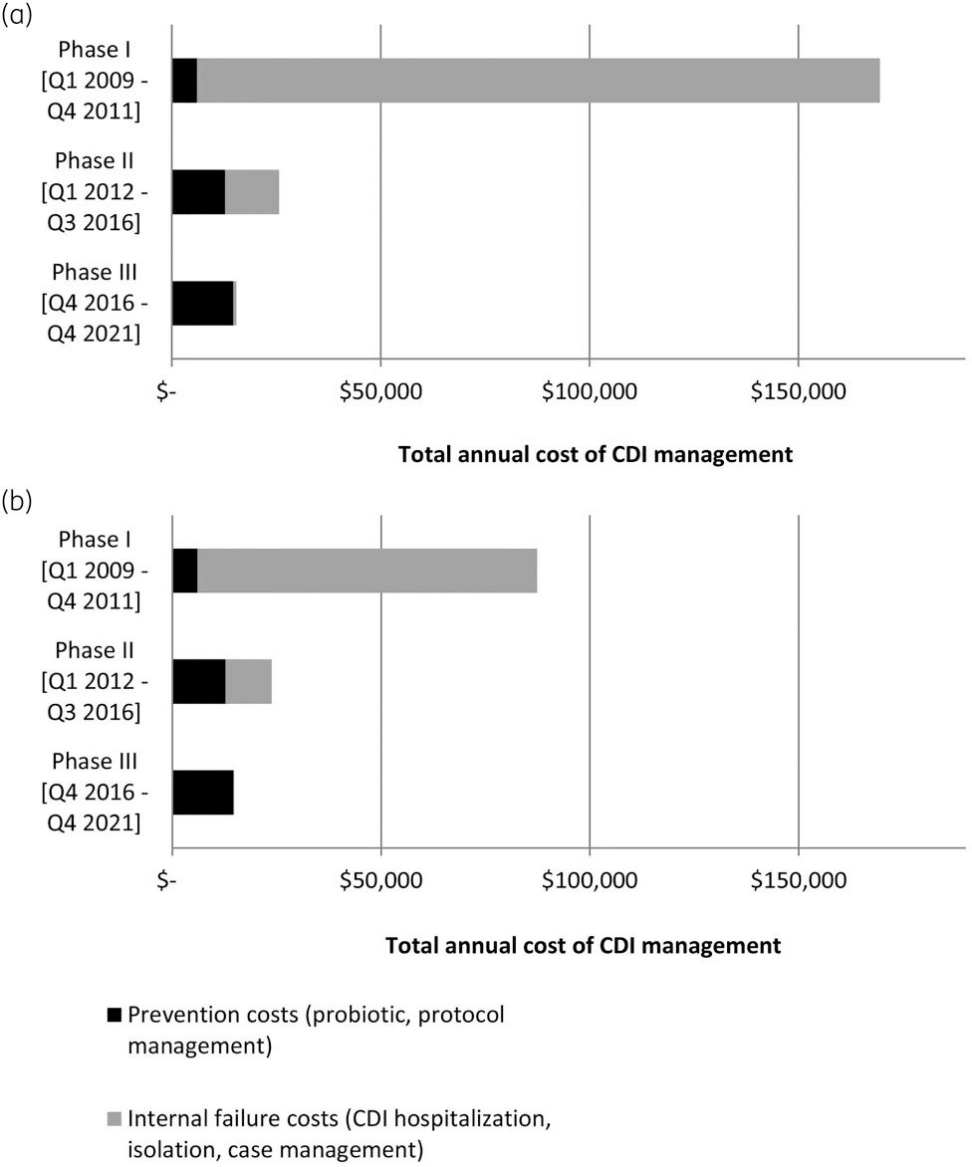
Two models to estimate the annual cost of CDI management among orally fed, probiotic-eligible patients considering both prevention and treatment costs. For model (a), an average daily cost of hospitalization is applied to both levels of care. For model (b), the daily cost of hospitalization is weighted by level of care (ICU versus medical-surgical unit).

A cost model was constructed to estimate the cost consequence, or Cost of Quality,^10^ of running a prevention programme that provides an oral probiotic to antibiotic users, taking the SNF’s perspective. Hospitalization costs (Table 1) related to CDI in this model are based on the actual number of days at the associated hospital’s acute care. Real costs to the facility were used when available, but some reference costs and rates were applied. The average daily cost of inpatient hospitalization is based on a reference cost for a non-profit hospital in California.^19^ (Figure 1a). As a sensitivity analysis, the cost of an inpatient day in intensive care was estimated at 1.5 times the reference cost, and the cost of an inpatient day in general acute care was estimated at 0.5 times the reference (Figure 1b).

**Table 1.** List of CDI-related cost estimates for testing, treatment, case management and administration of the probiotic protocol for orally fed patients

Variable	Cost estimate	Reference
Testing costs		
Laboratory charge, CDI test	$17.50	Actual
CDI care costs		
Inpatient day, non-profit hospital, California (2020)		
Average	$4464	^19^
Acute care (0.5 multiplier)	$2232	
ICU (1.5 multiplier)	$6696	
Treatment at theSNF	$1082 total/case	
Days of treatment for CDI case	8	^20,21^
Excess room cleaning/day	$24.68	^22^
Excess nurse time/day	$34.83	^22^
Additional isolation costs/day	$41.67	^22^
Gowns, gloves/day	$29.03	^22^
CDI antibiotics/day^a^	$4.76	Actual^22^
Infection prevention/ASP team costs (h)		
CDI episode management	4	Actual
Probiotic protocol management, monthly	1	Actual

a Vancomycin 125 mg every 6 h.

Purchasing and compliance data were not tracked for the liquid probiotic. Thus, only the cost-effectiveness of the oral probiotic was quantifiable.

### Statistical analyses

Data analysis was performed on the purchase records for the three-species *Lactobacillus* probiotic used from January 2012, to quantify the use of this probiotic preparation for primary and recurrent CDI prevention. The number of CDI cases and days of treatment required in acute care versus the SNF were calculated and compared for each phase of ASP policy changes affecting probiotic use. Statistical comparisons between groups were performed using discrete data in R statistical software (version 4.0.3; epitools) though some results are also reported as ratios or percentages. Rates and incidences were compared with Fisher’s exact tests in standard or modified two-by-two contingency tables.

### Research ethics

Patient records maintained by the ASP and probiotic records were de-identified. The plan for this retrospective study was reviewed and approved by the institution’s Internal Review Board (IRB) #2102807. The presentation of results follows the SQUIRE 2.0 (Standards for Quality Improvement Reporting Excellence) publication guidelines.^23^

## Results

### Treatment of CDI cases

There were 53 primary CDI cases and 19 recurrences at the LTC facility during the study period, 2009–21. More than half of the primary CDI cases occurred in enterally fed patients.

**Figure 2.**
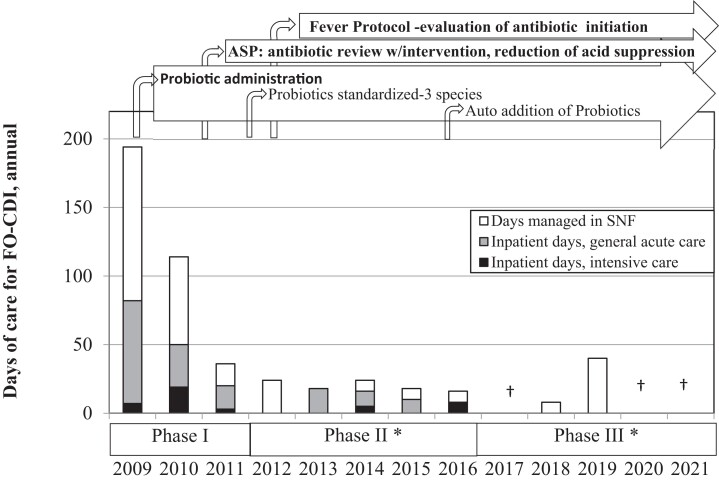
The number of days of care for primary and recurrent FO CDI cases spent in acute care, intensive care, or at the SNF each year among probiotic-eligible residents. With the difficulty in quantifying extra care required in patients not transferred from the SNF, each CDI case managed in the SNF was calculated as requiring an estimated 8 days of treatment.^24^*Significantly fewer total days of care and fewer days of inpatient care versus the previous phase, *P* < 0.05. †No CDI cases during the year.

The incidence of facility-onset (FO) CDI was 5.8 cases/10 000 patient-days in 2009, decreasing to an average annual rate of 0.3 in Phase III. Recurrence rates fell from 62% at baseline to 6% by Phase III. To aid in the cost–benefit analysis of the use of probiotics in different phases of policy implementation, we analysed the relative levels of care required for treatment of CDI. Of the 72 primary and recurrent CDI episodes, half were managed within the SNF and did not require acute hospital care. The highest number of inpatient (acute care) days required due to SNF-onset CDI was 152, observed in the 3 years of Phase I. There were significantly fewer inpatient days in later phases: 48 required for treatment of CDI complications in the 4.75 years of Phase II [OR = 0.22 (0.16–0.31)] and only 4 days in the 5.25 years of Phase III [OR = 0.08 (0.03–0.23)]. There have been no inpatient days required as a result of SNF-onset CDI since the end of 2016 (Figure 2).

### Probiotic use

Primary prophylaxis with a probiotic was frequently missed, increasing vulnerability for many of the CDI-positive patients in Phase I (65%) and Phase II (83%). During Phase III, when the ASP held the authority for probiotic prescribing, none of the eligible primary CDI cases lacked probiotics for prophylaxis. Probiotics were contraindicated for nil per os (NPO) patients not receiving enteral feedings (one primary CDI case and one recurrence in Phase II; two primary CDI cases in Phase III).

Probiotics were generally well tolerated across microbial strains and formulations, which were administered as capsules and tablets, in addition to liquid yogurt formulations for feeding tube administration. No *Lactobacillus* or *Saccharomyces* bacteraemias were reported or observed for patients taking probiotics.

The three-species *Lactobacillus* probiotic was prescribed to 770 orally fed patients from 2012 to 2021, with a median of age 78 years (IQR 65–87; range 20–102). The median duration of each order was 14 days, although patients with extended periods of antibiotic use and increased CDI risk took longer courses; 34 patients took the three-species *Lactobacillus* probiotic for 90 days or longer over the course of their residency.

### Cost modelling in orally fed patients

The modelled cost to treat a CDI episode anticipates testing, case management by an ASP member and the various daily expenses of the treatment setting, as outlined in Table 1. The modelled cost of managing a CDI episode at the LTC facility was $1337. The cost of a CDI episode requiring hospitalization was more than 10-fold higher at $18 113–$25 553.

The net cost savings associated with administering oral probiotics for primary prevention of CDI considered the costs of purchasing and administering the oral probiotic as well as the cost of treating and managing each CDI episode (Table 1). The facility-wide annual cost to purchase the oral probiotic increased from $6019 in Phase I to $12 726 and $14 652 in Phases II and III, respectively, due to both increased probiotic price and more consistent probiotic prescribing, (Figure 1a and b). Despite increased probiotic spending, there was a net decrease in the overall annual cost of CDI among orally fed patients (Phase I to Phase III, $72 544–$154 085 decrease). The incremental cost savings (Cost of Quality) was 9.8- to 21.4-fold greater than the incremental oral probiotic cost for Phase II, and 4.6- to 5.3-fold for Phase III (Figure 1).

## Discussion

Severe CDI disease and recurrences are more frequently found in SNFs.^25^ The cost consequences of nosocomial infections such as CDI are a major financial burden. In addition to the increased costs for care and treatment of CDI and its complications, the Centers for Medicare & Medicaid Services (CMS) imposes significant financial penalties for hospital readmissions, as an incentive to encourage SNFs to improve the quality of care they provide to Medicare beneficiaries. Additionally, hospital-acquired infections (HAIs) will soon be included as a cause for sequestration of funding.^26^ Therefore, it is important to identify cost-effective methods to prevent CDI in the LTC setting. Initially, our ASP policies promoted a decrease in the CDI rate and severity of complications in our patients, as evidenced by both a reduction in the days of treatment and transfers to acute care required (Figure 2). The automated addition of probiotics was initiated after other ASP processes were established and allowed for a further reduction in care needs and costs associated with CDI, with a highly favourable benefit to cost ratio (Cost of Quality 4.6–5.3).

Our cost model found a greater than 10-fold difference in the cost of managing a CDI episode that required hospitalization compared with management within the SNF. Policy changes that promoted the use of the probiotics resulted in fewer acute care days (Figure 2) and cost savings (Figure 1a and b). A portion of the antibiotic-treated patients received an order for a probiotic in Phases I and II, yet the majority of those who contracted CDI had missed this prophylaxis. The shift to an automated ordering protocol for probiotic prophylaxis in Phase III led to greater compliance, with no eligible CDI cases lacking probiotic prophylaxis in 5 years, 2017–21, with 3 of those 5 years having zero CDI cases.

The incremental costs of purchasing and administering the oral probiotic in Phase II and Phase III were minimal relative to the cost savings that resulted from avoided CDI cases and days in the acute care hospital. For this facility, the total annual cost of administering probiotics with all antibiotic courses was far less than the cost of treating a single CDI case in acute care.

The use of high-risk and multiple, concurrent antibiotic courses, or extensive durations of therapy, all can increase patient susceptibility to CDI.^8,27,28^ Many of our ASP actions allowed for a reduction of antibiotic pressure, making *C. difficile* spores less likely to take hold and infect the patient. Similarly, standardizing the addition of a three-species *Lactobacillus* probiotic^14,29^ with adequate dosing,^16^ alongside antibiotic treatment, anticipated the expected loss of intestinal microbiota,^6,17^ potentially reducing vulnerability to CDI. Our initial multipronged approach to CDI prevention yielded a significant drop in the number of days of care required for primary and recurrent CDI cases, by the end of Phase I. Concurrent ASP interventions with optimization of antibiotic prescribing and reduction of unneeded acid suppression, initially made it difficult to separate out the impact of the addition of probiotic therapy. However, with ASP processes remaining consistent from 2013 on, CDI severity continued to decrease with the more automated addition of probiotics to antimicrobial therapy in Phase III. Our study is consistent with results found in other studies adding this probiotic as an automated part of ASP efforts to reduce CDI. Maziade *et al*.^24^ found the incidence of hospital-acquired CDI was lower by at least half for patients who took the same three-strain *Lactobacillus* probiotic with multiple-antibiotic regimens, or regimens that included high-risk antibiotics. Their pharmacy-driven protocol allowed for a more widespread implementation of the probiotic (75%), compared with a similar study that did not find probiotics to be a benefit but had only 17% of eligible patients with probiotic implementation through their electronic flagging system.^30^

After a long hiatus from CDI in our SNF units (seven quarters), a CDI cluster of three cases occurred in 2019 (Phase III) with patients in rooms of close proximity, following a lack of prompt isolation of a newly admitted, symptomatic CDI patient. The cluster was determined to be an infection prevention issue and education was done to reinforce established policies. This was a reminder that ongoing staff education and training is required to keep practices current. The extraordinary reinforcement of all infection control measures during the 2020–21 COVID-19 pandemic was shown to limit the nosocomial spread of *C. difficile* during those years.^31^ Although we had no CDI cases during 2020–21, our sustained reduction in nosocomial cases started prior to that time and has continued to the present.

Antibiotic prescribing must be optimized through a combination of ASP policies and practices, in addition to consistent infection prevention processes, to effectively minimize CDI risk. Efforts to limit antibiotic pressure at this SNF and the affiliated hospital resulted in an initial 30% decrease in antibiotic use by 2014.^12^ However, a change in the case-mix of residents allowed for an increased proportion with morbidities requiring antibiotic treatment, which made later antibiotic use comparisons difficult after 2014, as we were unable to show further decreases in overall antibiotic use. Yet, the number of FO CDI cases and days requiring acute care treatment continued to decline.

Future studies on the use of probiotics for primary prevention of CDI in SNFs should anticipate selecting the most efficacious probiotics and the challenges of reaching a high proportion of both orally and enterally fed patients.

### Conclusions

It was safe and beneficial to implement probiotic prophylaxis of CDI for antibiotic users in the SNF. Optimizing probiotic use by selecting an evidence-based probiotic combination, with a policy allowing for ASP-ensured compliance, brought continued decreases in costs associated with CDI and reduced the need for acute care hospitalizations in probiotic-eligible patients, after other ASP processes were established.
